# The issue of ‘informed consent’ in medical student introductions

**DOI:** 10.1007/s40037-015-0170-3

**Published:** 2015-03-31

**Authors:** Rahul Prashanth Ravindran, David George Lester, Khizr Ather Nawab, Sayinthen Vivekanantham

**Affiliations:** Imperial College School of Medicine, Imperial College London, London, SW7 2AZ UK

‘Good morning! My name is John Smith and I am a medical student.’ This familiar phrase is employed by medical students the world over in a variety of formats. Despite the varying terminology, the purpose of this introduction remains to obtain ‘informed consent’ from patients. Even though the importance of informed consent is universally acknowledged by doctors, patients and students, Silver-Isenstadt and Ubel [1] found evidence for a worrying decline in the perceived importance of conveying one’s student status in medical students as they progress through medical school. This was in contrast to patients’ desires for clear communication of medical student roles and the scope of their involvement, especially in the context of invasive procedures in operating theatres under the influence of anaesthesia [1]. This calls into question the adequacy of current medical student introductions in obtaining true informed consent.

Recently, Carson-Stevens et al. [2] surveyed medical students’ perceptions on the role of terminology used for introducing themselves and found that students knowingly employed varying terminology, often choosing to introduce themselves as ‘student doctors’. Students perceived this to have a dual benefit of increasing the probability of patient consent and reassuring patients of the near-professional status of the student [2]. However, Santen et al. [3] confirmed that even when informed of the medical student’s relative inexperience, 90 % of patients consented to a procedure in the emergency department although 66 % of patients did feel that they should be informed if the medical student is performing the procedure for the first time [3].

We believe that the continued use of ambiguous introductions by medical students restricts patient autonomy by withholding essential information, and this also erodes trust in the student/doctor-patient relationship. We propose that medical students approach the matter of clinical introduction by introducing themselves unambiguously as medical students, explaining their affiliation with the medical team and indicating the breadth of their experience prior to requesting consent.

## References

[1] Silver-Isenstadt A, Ubel PA (1999). Erosion in medical students. J Gen Intern Med.

[2] Carson-Stevens A, Davies MM, Jones R, Chik ADP, Robbé IJ, Fiander AN (2013). Framing patient consent for student involvement in pelvic examination: a dual model of autonomy. J Med Ethics.

[3] Santen SA, Hemphill RR, Spanier CM, Fletcher ND (2005). ‘Sorry, it. Med Educ.

